# Supplementary material for: Gene silencing for invasive paper wasp management: Synthesized dsRNA can modify gene expression but did not affect mortality
Source: PLoS One. 2023 Jan 3;18(1):e0279983. doi: 10.1371/journal.pone.0279983 (PMC9810182; doi:10.1371/journal.pone.0279983)
Supplement: S1 Table — (PDF) [file pone.0279983.s001.pdf]

S1 Table. List of genes whose expression was targeted in *Polistes dominula* foragers with double-stranded RNA (dsRNA) to assess mortality in this study.

Target #	Gene name (abbreviation)	NCBI ID for *Trilobium** homolog	Blast sequence with 100% identity in *P. dominula*	Pathway involved	Reference
**1**	DNA-directed RNA polymerase II subunit RPB2 (RpII140)	XM_969560.4	XM_015319188.1	DNA-dependent RNA polymerase that catalyses the transcription of DNA into RNA.	Knorr et al. (2018)
**2**	FACT complex subunit spt16 (DRE4)	XM_967384.3	XM_015322365.1	Nucleosome reorganisation for transcription.	Knorr et al. (2018)
**3**	CWC22 homolog (NCM)	XM_001811253.3	XM_015332511.1	Required for pre-mRNA splicing as component of the spliceosome.	Knorr et al. (2018)
**4**	Ras opposite (ROP)	NM_001170684.1	XM_015331750.1	Secretion / synaptic transmission.	Knorr et al. (2018)
**5**	DNA-directed RNA polymerase II subunit RPB7	XM_965220.3	XM_015320902	DNA-dependent RNA polymerase catalyses the transcription of DNA into RNA. RPB7 is part of a subcomplex with RPB4.	Knorr et al. (2018)
**6**	Putative U5 small nuclear ribonucleoprotein 200 kDa helicase (BRR2)	XM_965461.3	XM_015330523.1	Plays role in pre-mRNA splicing as core component of precatalytic, catalytic and postcatalytic spliceosomal complexes.	Knorr et al. (2018)

Target #	Gene name (abbreviation)	NCBI ID for *Trilobium** homolog	Blast sequence with 100% identity in *P. dominula*	Pathway involved	Reference
**7**	Kinesin-like protein KIF11-A (Klp61F-1)	XM_008194324.2	XM_015332401.1	Unknown/undetermined.	Knorr et al. (2018)
**8**	Tubulin α-1 chain (α-TUBULIN)	KX228233.1<br>**Blatella germanica*<br>**Varroa destructor*	XM_015323874.1	Tubulin is the major constituent of microtubules.	(Garbian et al., 2012; Huang et al., 2018)
**9**	NF-kappa-B inhibitor cactus (CACT)	NM_001163711.1	XM_015325572.1	Part of the Toll signalling pathway which is activated following infection with fungi or Gram-positive bacteria.	Bingsohn et al. (2017)
**10**	CACTIN	XM_008197782.2	XM_015324777.1	A poly(A) RNA-binding protein. It interacts with *cact* in early embryonic development. Involved in the dorsal-ventral embryonic patterning. Probably acts as a negative regulator of the NF kappa-light-chain-enhancer signalling pathway.	Bingsohn et al. (2017)
**11**	RNA-binding protein fusilli (FUSILLI)	XM_965708.4	XM_015327754.1	The mRNA splicing factor encoded by *fus* is involved in embryonic dorsoventral patterning and is expressed in the ovaries during later embryonic development. The loss of *fus* activity during embryogenesis is lethal.	Bingsohn et al. (2017)
**12**	Charged multivesicular body protein 4b (DVSNF7)	XM_028287710.1<br>**Diabrotica virgifera virgifera*	XR_001475970.1	Probable core component of the endosomal sorting required for transport complex III (ESCRT-III) which is involved in multivesicular bodies (MVBs) formation and sorting of endosomal cargo proteins into MVBs. MVBs contain	Bolognesi et al. (2012)

Target #	Gene name (abbreviation)	NCBI ID for *Trilobium** homolog	Blast sequence with 100% identity in *P. dominula*	Pathway involved	Reference
				intraluminal vesicles that are generated by invagination and scission from the limiting membrane of the endosome and mostly are delivered to lysosomes enabling degradation of membrane proteins, such as stimulated growth factor receptors, lysosomal enzymes and lipids.	
**13**	Anthonomus grandis chitin synthase II (AGRA)	KF147150.1<br>**Anthonomus grandis*	XM_015336156.1	Catalyses the final biosynthesis step of chitin. Functional analyses using RNAi in different insect species show that ChSs are required for survival, ecdysis, oviposition and egg hatching.	Macedo et al. (2017)
**14**	Similar to 26S proteasome non-ATPase regulatory subunit 6 (RPN7)	XM_968550.1	XM_015325431.1	Structural complex; genetic information processing; proteasome.	Knorr et al. (2018)
**15**	Fork head (FKH)	NM_001039414.2	XM_015327321.1	Longevity regulating pathway in multiple species. In yeast, the transcription factors FOXO, DAF-16, Gis1, and Msn2/4 transactivate genes involved in resistance to oxidative stress, energy metabolism, DNA damage repair, glucose metabolism, autophagy and protection of proteins by chaperones.	Knorr et al. (2018)
**16**	Calmodulin-like (CaM)	XM_022799184.1<br>**Varroa destructor*		Calmodulin (CaM) is an essential calcium-binding protein that regulates multiple protein targets.	(Inberg & Mahak, 2016; Krishnan et al., 2021)

Target #	Gene name (abbreviation)	NCBI ID for *Trilobium** homolog	Blast sequence with 100% identity in *P. dominula*	Pathway involved	Reference
**17**	DNA-directed RNA polymerase I subunit RPA1-like (RPA1)	XM_022807260.1<br>**Varroa destructor*	XM_015325844.1	RNA polymerase beta subunit. RNA polymerases catalyse the DNA dependent polymerization of RNA. Each RNA polymerase complex contains two related members of this family, in each case they are the two largest subunits. The clamp is a mobile structure that grips DNA during elongation.	(Garbian et al., 2012)
**18**	DNA-directed RNA polymerase III subunit RPC2-like (RPC2)	XM_022831563.1<br>**Varroa jacobsoni*	XM_015323394.1	RNA polymerase beta subunit. RNA polymerases catalyse the DNA dependent polymerization of RNA. Each RNA polymerase complex contains two related members of this family, in each case they are the two largest subunits.	(Garbian et al., 2012)
**19**	V-type proton ATPase 116 kDa subunit a-like (VATPase)	XM_022792920.1<br>**Varroa destructor*	XM_015316313.1	The V-type ATPases family are proton pumps that acidify intracellular compartments in eukaryotic cells for example yeast central vacuoles, clathrin-coated and synaptic vesicles. They have important roles in membrane trafficking processes. The 116kDa subunit (subunit a) in the V-type ATPase is part of the V0 functional domain responsible for proton transport. The a subunit is a transmembrane glycoprotein with multiple putative transmembrane helices it has a hydrophilic amino terminal and a hydrophobic carboxy terminal. It has roles in proton transport and assembly of the V-type ATPase complex.	(Garbian et al., 2012)
**20**	Sodium/potassium-transporting ATPase subunit beta-2-like (NaKATPase)	XM_022832680.1<br>**Varroa jacobsoni*	XM_015333664.1	Na+/K+ATPase is an integral membrane protein responsible for establishing and maintaining the electrochemical gradients of Na and K ions across the plasma membrane. These gradients are essential for osmoregulation, for sodium-coupled transport of a variety of organic and inorganic molecules, and for electrical excitability of nerve and muscle. This enzyme is composed of two subunits, a	(Garbian et al., 2012)

Target #	Gene name (abbreviation)	NCBI ID for *Trilobium** homolog	Blast sequence with 100% identity in *P. dominula*	Pathway involved	Reference
				large catalytic subunit (alpha) and a smaller glycoprotein subunit (beta).	
**21**	Apoptosis inhibitor 5-like (APOPIN)	XM_022790626.1<br>**Varroa destructor*	XM_015325067.1	Apoptosis or programmed cell death is a physiological form of cell death that occurs in embryonic development and organ formation. It is characterized by biochemical and morphological changes such as DNA fragmentation and cell volume shrinkage. API5 is an anti-apoptosis gene located in human chromosome 11, whose expression prevents the programmed cell death that occurs upon the deprivation of growth factors.	(Garbian et al., 2012)
**22**	Putative inhibitor of apoptosis (INAPOP)	N/A	XM_015333547.1	Composed of three domains. First, baculoviral inhibition of apoptosis protein repeat domain found in inhibitors of apoptosis proteins (IAPs) and other proteins. Second domain found in inhibitor of apoptosis proteins (IAPs) and other proteins. Acts as a direct inhibitor of caspase enzymes. Third domain is a Zinc finger, C3HC4 type.	This study

References

Bingsohn, L., Knorr, E., Billion, A., Narva, K. E., & Vilcinskas, A. (2017). Knockdown of genes in the Toll pathway reveals new lethal RNA interference targets for insect pest control. *Insect Molecular Biology*, *26*(1), 92-102. https://doi.org/doi:10.1111/imb.12273

Bolognesi, R., Ramaseshadri, P., Anderson, J., Bachman, P., Clinton, W., Flannagan, R., Ilagan, O., Lawrence, C., Levine, S., Moar, W., Mueller, G., Tan, J., Uffman, J., Wiggins, E., Heck, G., & Segers, G. (2012). Characterizing the mechanism of action of double-stranded RNA activity against western corn rootworm (*Diabrotica virgifera virgifera* LeConte). *PLOS One*, *7*(10), e47534. https://doi.org/10.1371/journal.pone.0047534

Garbian, Y., Maori, E., Kalev, H., Shafir, S., & Sela, I. (2012). Bidirectional transfer of RNAi between honey bee and Varroa destructor: Varroa gene silencing reduces Varroa population. *PLoS Pathog*, *8*(12), e1003035. https://doi.org/10.1371/journal.ppat.1003035

Huang, J. H., Liu, Y., Lin, Y. H., Belles, X., & Lee, H. J. (2018). Practical use of RNA interference: Oral delivery of double-stranded RNA in liposome carriers for cockroaches. *Journal of Visualized Experiments*(135), e57385. https://doi.org/doi:10.3791/57385

Inberg, A., & Mahak, K. (2016). *U.S. Patent 62156751*. B. I. Monsanto Technology LLC.

Knorr, E., Fishilevich, E., Tenbusch, L., Frey, M. L. F., Rangasamy, M., Billion, A., Worden, S. E., Gandra, P., Arora, K., Lo, W., Schulenberg, G., Valverde-Garcia, P., Vilcinskas, A., & Narva, K. E. (2018). Gene silencing in *Tribolium castaneum* as a tool for the targeted identification of candidate RNAi targets in crop pests. *Scientific Reports*, *8*(1), 2061. https://doi.org/10.1038/s41598-018-20416-y

Krishnan, N., Hall, M. J., Hellmich, R. L., Coats, J. R., & Bradbury, S. P. (2021). Evaluating toxicity of *Varroa* mite (*Varroa destructor*)-active dsRNA to monarch butterfly (*Danaus plexippus*) larvae. *PLOS One*, *16*(6). https://doi.org/10.1371/journal.pone.0251884

Macedo, L. L. P., Antonino de Souza Junior, J. D., Coelho, R. R., Fonseca, F. C. A., Firmino, A. A. P., Silva, M. C. M., Fragoso, R. R., Albuquerque, E. V. S., Silva, M. S., de Almeida Engler, J., Terra, W. R., & Grossi-de-Sa, M. F. (2017). Knocking down chitin synthase 2 by RNAi is lethal to the cotton boll weevil. *Biotechnology Research and Innovation*, *1*(1), 72-86. https://doi.org/https://doi.org/10.1016/j.biori.2017.04.001
