# Supplementary material for: Gene silencing for invasive paper wasp management: Synthesized dsRNA can modify gene expression but did not affect mortality
Source: PLoS One. 2023 Jan 3;18(1):e0279983. doi: 10.1371/journal.pone.0279983 (PMC9810182; doi:10.1371/journal.pone.0279983)
Supplement: S2 Table — (PDF) [file pone.0279983.s002.pdf]

S2 Table. Sequences for the 22 dsRNAs tested in *P. dominula* in this study and the qPCR primer pairs used to determine expression of the targeted genes.

**Gene target and dsRNA sequence**	**Forward primer name**	**Forward primer sequence**	**Reverse primer name**	**Reverse primer sequence**	**Product length (bp)**	**Comments**	**Reference**
**PROD54**	PD-<br>PROS54F	ACTCTTACAAGCG<br>ATGTTGGT	PD-<br>PROS54R	CTGTCGATGCTTTAGGGCC<br>A	117	Internal reference gene 1	Cameron et al. (2013)
**NDUFA38**	PD-<br>NDUFA38F	TGACGGCTTGTGGT<br>ATGGAA	PD-<br>NDUFA38R	TCGACACTGGCTAAACTCC<br>A	119	Internal reference gene 2	Cameron et al. (2013)
**RPII140**<br>TAGTGCAGTTGAGAGAG<br>GTTTCTTTAGATCTGTCTT<br>TTACCGATCTTATAAGGA<br>TTCTGAATCTAAAAAAAT<br>TGGTGATCAGGAAGAAC<br>AATTTGAAAAACCTACAA<br>GACAAACTTGCCAAGGA<br>ATGCGCAATGCTATATAC<br>GATAAATTAGACGATGAT	PD-RPII140<br>A 2311 F	AAGTCCTAGGAAT<br>ACTTATCAG<br>AGTGC	PD-<br>RPII140-A-<br>2410R	AAGTACGTGAGCTAAAG<br>TGTCCA	100		This study
**DRE4**<br>AGACGATGAAGAAGAAG<br>AGGAAGAAGGCAGTGGC<br>AAAGAAAACGAGCCTAA<br>ACCTGAGATATTGGGTAG<br>AGGTAAAAGAACAGCTG<br>TCATAGAATCCAAGTTAA<br>GAACGGAACATAGTTCA<br>GAGGAAAAGAGAAAACA<br>ACATCAAAAAGAGTTAG	PD-DRE4-<br>A-1396F	TCCAGATGCTACTG<br>AGAAAGA<br>AGG	PD-DRE4-<br>A-1475R	CAGGTTGACCCTCATTGAC<br>CA	80		This study

CTCAACAGTTGAATGAAATTGCTAAAGCTCGATTGGCTCAACAATCTGGAGGAAAAGAACAAGAAAAAATACGTAAATCAACTGTCTCATATAAGAGTTTAAACCATATGCCACGTGAACCAGAAGTGAAAGAACTAAAGTTATATGTTGATAAGAAGTACGAAACAGTAATTTTACCAATTTTTGGAATTCCCGTACCATTCCATATATCAACAATCAAGAACATTTCTCAATCTGTTGAGGGTGACTATACGTATCTTAGAATAAATTTCTTTCATCCTGGTGCAACAATGGGTCGTAACGAAG							
**NCM**<br><br>CGGAUAGACGUUCAGAAAGAUAUUCGGAUAGACGUUCAGAAAGAUAUUCGGAGAGACGCUCAGAUAGGCGUUCGGAAAGACGUUCAGAAAGACGUUCAGAUAAACAUUCAGAUAAACAUUCGGAUAAACAUUCGGAUAAACAUUCGGAUAAAUAUUCGGAUAAAUAUUCGGAUAAAUAUUCGGAAAGAAAAACUACAACAGAAACAAUGCCGGAGAAUCGGAAAAGGACUCAUGAUGUAUUAACAGCAAAAACAGGAGGUGCUUAUAUACCA	PD-NCM-F	GGGAAGAGGTTTGTTAGCTCGT	PD-NCM-R	AGTGCTGCATAAACAGGAGTGA	78		This study

CCAGCAAAAUUGCGUAU GAUGCAAGCAAAUAUCA CGGACAAGUCUGGGGCU GCAUAUCAGCGGAUUGC AUGGGAAGCA							
**ROP** AGAAGUGACUUUGACCG CAACGUGGAAUUGGCAC AGAUGGUUCAACAGAAA UUGGAUGCUUAUAAAGC AGAUGAACCUACUAUGG GAGAAGGUCCAGAAAAA GCUCGCUCUCAAUUACU AAUCCUAGACAGAGGUU UUGAUUGUGUUUCUCCC UUGCUUCAUGAAUUGAC AUUACAGGCUAUGGCAU ACGACUUGUUGGAUAUA GACAAUGAUGUUUACAG AUUCGAAGCAUCUGCUG GCGUGGAAAAAGAAGUU CUUUUAGAUGAAAACGA CGAUUUAUGGGUAGAAU UAAGACAUCAACACAUU GCUG	PD-ROP-F	AACTCTGCAAGTCT CTCGCC	PD-ROP-R	ACTCATAGGGCAGGAAAG CG	75		This study
**RPB7** UAGUUAAACAAAAACUU UAUACAGAAGUUGAAGG AACAUGUACUGGAAAGU AUGGCUUUGUUAUAGCA GUAACACUUAUAGAUAA UAUCGGUGCUGGUAUAA UACAACCAGGAGAAGGU UUUGUUGUUUAUCCUGU	PD-RPB7-F	TGGACCTCAACTGC TTGAAA	PD-RPB7-R	ACAAAGCCATACTTTCCAG TACA	81	Primers necessarily overlap the dsRNA region	This study

UAAAUAUAAAGCUAUUG UAUUUAGACCAUUUAAA GGUGAAGUUUUGGAUGC AAUUGUAACACAAGUAA ACAAGGUUGGAAUGUUU GCAGAAAUAGGACCAUU AUCUUGUUUUAUAUCUC AUCAUUCAAUACCAGAA GAAAUGCAAUUUUGUCC CAAUGUUAAUCCACCAU GUUACAAAUCAAAAGAA GAGGACGUGAUCAUUCA AGCUGAUGAUGAAAUAA GAUUAAAAAUAGUUGGA AC							
**BRR2** GCACCAGCUGCUGCAAC UUUUAACUUUCAUCCUU CUGUUAGACCAGUACCA UUGGAAUUACACGUUCA AGGCAUUAACAUUACUC AUAAUGCAUCCAGACUG GCUGCUAUGGCUAAACC AGUGUAUAAUGCGAUAC UUAGGCAUGCUUCUCAC AAACCUGUUAUUGUCUU UGUGCCGACUCGUCGAC AAGCCAGAUUAACAGCU AUCGAUUUAUUAACAUU UACAGCAGCAGAAGGUG CACCAUCAAGAUUUUUC CAUGCUGAAGAAGCUGA CAUCAAACCUUUCUUAG AUAGAAUGACAGAUAAA ACAUUGAAAGAGACCUU	PD-BRR2-F	ACACCAGAAAAGT GGGACATCA	PD-BRR2-R	ACCTCTTTCGTCGTGCAAC A	111		This study

GUCUCAAGGUAUAGCUU AUUUGCAUGAAGGACUU UCUGCAGAUGAUCGGCG UUUAGUUGAACAAUUGU UUGACAGUGAUGCCAUU CAAGUAGCAGUGGCAAC CAGAGACUUGUGUUGGA GUUUAUCCAUCAGUUCA CAUCUGGUAGUAGUAAU GGACACUCAAUGUUACA AUGGUAA							
**KLP61F-1** UAAACCUGAAAGAAAUG AGGACCAUAAUAUUGAA AUAAUAAUGAAACAAGC UGCAGAACAACUUAAUC AGGGAUCAAUUACAAAA ACGCAAUACAAUACGUU AAUUCAAGAAGUUUUAC AUAUGAGUGAAGAUCGA AAAUUGAGAGCAGCACA GCGUAAAGAGAAAGAAG UAGGUUCUAUAGUAUGG GAAAAAGGAGUGACAUU AGAUAUUAAUACAAUUG AUCGUGGAGCUGCUUUU AGUCCAACGAAAGAUAA AGAAGUAAUAAGAAAUA AGGAUCAUCCUAUUCCU AGAAAUCCAAAUGGUCC ACGAUGGCAAGGCCAGC CAUGGCAGCAACCAGGA CCAUGGGCUCAUCCACC UGGUCCACCUUAUGGUG GUCCUCAAGCACACUUU	PD-KLP6-F	ACGAATTGCACCA GCGAGTA	PD-KLP6-R	CTCCTGAACGGTTGCAGTC T	88		This study

AAUGCAGAUUUUAGACC UGUAGGGCCUUGGCAAA AUCCAAGACACUUUGGC CCAAUGAGACCAGACUA UCAGUAUCACGGUUAUA GCCACAAUAUGGCUCCC AAUCCUC							
**α-TUBULIN** CAUACCACGCUUGAACA UUCCGACUGCGCAUUUA UGGUUGACAACGAAGCU AUUUAUGAUAUUUGUCG UCGUAAUUUGGACAUCG AAAGACCAACAUACACC AAUUUGAAUCGUUUGAU CGGUCAAAUCGUUUCUU CAAUCACUGCAUCUCUA CGAUUCGAUGGUGCUCU CAAUGUCGACUUAACGG AAUUCCAAACUAACUUG GUACCUUAUCCAAGGAU UCAUUUCCCAUUGGUAA CUUAUGCACCGGUCAUA UCUGCGGAAAAGGCUUA CCAUGAACAAUUAAGCG UAGCUGAAAUUACUAAU GCUUGCUUCGAACCAGC CAAUCAGAUGGUCAAAU GCGAUCCACGACACGGA AAAUACAUGGCCUGCUG UAUGUUGUACAGAGGUG AUGUCGUACCAAAGGAU GUUAAUGCCGCUAUUGC UACCAUCAAAACUAAAC GCACGAUUCAAUUCGUU	PD-aTUB-F	ATTGGAGGTGGCG ATGACAG	PD-aTUB-R	TGCGGACCTCATCAACAAC A	115		This study

GACUGGUGUCCGACCGGGUUCAAAGUUGGUAUCAAUUACCA							
**CACT**<br><br>AGAAGAAUUGGAAGAAAGAAACUGAGGAUUCGGAUCGCAUUGAUUCUGGAUUUCUUUCCAGUGGAAAUUUGCAACUCAGCUCCGAGCUCGAACGCUCGGAGUUGGACGUUGCAGGAGAUUUCAGUGUCGUAAAAACGGAACCAAUGAGAGCUGACAGUGGCGUAGAUGUUGGCCUAAGCGAAAGCUUGAGCCAACUGUCCCUCAAACAAGUUACUUUGAAUCCUUUAGCAAGUGGAAAAGUACAAGUCGAGCCUAUCACUGAACUUGCACCUCUAAGCCCAGAUCAACAAAAUAUUAUUAAUCAACAACAACAUCAUCAAGGAGACUAUGCAUCCCCAAUCAUUGACAAUAAUAUUGAAAUUCGUGAAAAUCGACAAAAAAGAUUUAUCGAGGAGCCUUGGCAGCUUUAUUACACGCAGGAUAAUGAAGGAGACACGCAACUACAUAUCGCCAUUGUACAAGGCUUUUUGGAGGCCGCCUUUUGUCUCAUAAGAAUGGCA	PD-CACT-F	TGCAGGCCTTGTTTGAATGC	PD-CACT-R	CCCAAGTCTGACCAGTTCCC	102		This study

**CACTIN**<br><br>AAUAUGAAGUAGCUGUU<br>GGUAGAAGAGAAGGAAU<br>UCAUUCGUCAGUGGCAA<br>AAGAUGUUACUGCUAUU<br>UUUAAAGGAAAAACAGC<br>AGCUCAGCUAGAAUCGC<br>UGCAAUUACAGAUUGAA<br>UCGAAAAUAACCGGGAA<br>ACCAGAAGGCGUUGAUA<br>UCGGUUACUGGGAAAGU<br>CUGUUGUCACAAUUAAA<br>AGCUCAUAUGGCAAGAG<br>CUAGACUAAGGGAUCGA<br>CAUCAAGAGAAUUUACG<br>UAAAAAAUUGGAAGUCU<br>UGAUUGCUGAACAGGGU<br>GUAGCUAGAACAGAAAG<br>UGAAGCAGAAGGAUCUC<br>AAGUACCAAGCGAGCAU<br>UCAACAAAACAGGAAGA<br>ACCUACUACGAGUAAUA<br>UCGAAAAAAGUGAAGAA<br>AGUCAAUCAGAGGACGA<br>ACAAGAAAAUGGGAAUG<br>CAGCAGAUGAUAUGUUA<br>UCUGAAUCAUUUUGUGA<br>AUAUGAAACAGGUGGCU<br>AUUCACCAAAAUAUAUA<br>CCUUACUCUCAACUGGA<br>ACCAGGA	PD-<br>CACTIN-F	GAACAGTGGGCGA<br>GACAAGA	PD-<br>CACTIN-R	CGGTTTAGCACGACCATCC<br>T	90		This study
**FUSILLI**<br><br>ACACUUGGAAUACAAGA<br>AAUGAUUCAAUAUUUUG<br>GGCUACAACCUGAUACG	PD-<br>FUSILLI-F	TACTATCACCCGGA<br>GGCACA	PD-<br>FUSILLI-R	GTGGTCCTCGTGGTAGCAT<br>C	114		This study

GACAACGAUUAUCAUGU CAAAGAAAUACAAGACA UGAUUAGCGUGAUACAG AGGAUGAUCAAAGAUGG UCACGUCUUCCAAACAC CAGAGAUAGUAAAUAUA GUUUUAGAACCAGGAAU AUGUUCCAAGGACGAAG AAGUAGACAACGAUUGC GUAGUUAGAGCAAGAGG CCUUCCUUGGCAAUCAU CGGAUCAAGAUAUUGCC AAAUUUUUCCGUGGUCU GAAUGUCGCAAAGGGUG GCGUAGCACUUUGUUUG AGUCAUAUGGGCAGGCG UAACGGAGAAGCUUUAG UACGCUUUGUUAAUAAA GAACACAGAGAUAUGGC ACUGAAAAGGCAUAAAC AUCAUAUGGGAGCACGG UACAUAGAAGUAUAUAA AGCUUCUGGAGAAGAUU UUGUUGGGGUUGCUGGU GGUACUACUAGCGAAGC GCAUGCUUUCCUUUCAA GAGGGGC							
**DVSNF7**<br><br>AAGAAACAAGACUUUCU UGAAAGCAAAAUUGAAC UUGAAAUUCAAACAGCG AAAAAAAAUGGAACGAA GAACAAGAGAGCGGCUA UCCAAGCUCUCAAAAGA AAAAAGCGUUAUGAAAA	PD-DVSNF7-F	AAATTGCGTGAAACCGAGGA	PD-DVSNF7-R	GCCGCTCTCTTGTTCTTCGT	113	Primers necessarily overlap the dsRNA region	This study

GCAGCUACAACAAAUCG AUGGUACUUUGUCAACG AUUGAAAUGCAAAGAGA AGCUCUGGAAAGUGCCA ACACAAAUACUGCGGUA CUUACCACCAUGAAAAG CGCAGCGGAUGCAUUAA AAUCUGCUCAUCAACAC AUGGACGUCGAUCAAGU UCACGAUAUGAUGGAUG AUAUUGCUGAACAACAA GAUGUUGCUAAAGAGAU UUCUGAUGCAAUAUCUA AUCCUGUAGCAUUUGGU CAAGAUAUAGAUGAAGA AGAACUUGAAAAAGAAC UAGAAGAACUUGAACAG GAACAACUUGAUAAAGA AUUACUUGGUAUCGAAU CUACAGAUGAAUUACCA GCAAUACCUGCUACAAC UGUUCCAGCUGUUCCAU CUGCUCC							
**AGRA**<br><br>GUGAAGAUCCAGCCGAG GAUGAAGAAUGUAACAG UGAAACAGAAACGGUAA CAAGCCAACAUUCGGAA GAAAAUCGUGAGGGUAG UAACUUCCUCACAAGAC CUUAUUGGUUGAGCGAC GAAGGAUUGAAAAAAGG UGAAGUGGAGAUCUUGU CACUUCAAGAAGAACAG UUUUGGAAAGAUUUACU	PD-AGRA-F B	TGGCAAATTCGCGTGCAAAA	PD-AGRA-R B	GCGTAGACCACAAGCAGCTA	109	2nd generation primers	This study

UGAAAAGUACCUCUUUC CAAUAGACGAAGACAAA GCGGAAAAGGCACGAAU CGCUAAAGAUCUAAAGG ACCUACGCGACCAGAGC GUGUUUGCUUUCUUUAU GCUGAAUGCCCUCUUCG UCCUGAUCGUCUUUUUG CUCCAACUAAACAAAGA CUUAUUACACGUGAAAU GGCCAUUUGGUAUCAAG AUGAACAUCACCUAUGA UGAAUACUUGCAAGAGG UACACGUUACCAAAGAA UACUUGCAACUUGAGCC UAUUGGUCUGGUGUUCG UGUUCUUUUUUGCAUUA AUUCUAGUCAUUCAAUU CACUGCU							
**RPN7** UAUUAAGUCUUCCGGAA CACCAUGAAGAUCCCUA UUUGAAAACUAAACUAC UCGAUGCCAUUAAAGCA GAAAAUAUGGCACCAUU UUACGAAGAAGUUUGUC GAGAUCUGGGUUGGAUA GUCGACGAAACGCUUUU GGCCAAUAUGAGAGCUC GCAAUGCGGAGCAAUUA AAAGAGUUAGAUGGAGC GAUUGAGGACGCUGAAA AGAAUUUAGGAGAAAUG GAAGUACGCGAAGCUAA UCUUAAAAAAUCCGAAC	PD-RPN7-F	ATGGCAGAAGCAT TTGGAGT	PD-RPN7-R	CGCCAACGCGATCAACTTT A	100		This study

AUCUUUGUCGAAUUGGG GACAAAGAAGGAGCGAU AACAGCUUUUAGAAAAA CUUAUGAUAAAACUGUG UCUUUGGGUCAUAGACU AGAUAUCGUAUUUCAUA AUAUUAGAAUUGGUUUG UUUUAUUUGGAUCACGA UCAUAUAACAAGGAAUA UAGAAAAAGCAAAAAGU UUAAUCGAAGAAGGAGG UGAUUGGGAUAGGCGAA AUCGCUUGAAAGUAUAU CAAGGAACUUAUUGUAU AGCAGU							
**FKH**<br><br>CGCCUCGUUACGAGAAA CCACCCUAUUCUUACAU AGCUUUAAUAGCCAUGG CUAUUAAUUCGUCACCG AAACAAAGACUCACUCU AUCCGGUAUUUACAAGU UCAUCAUGGAGAGGUUC CCUUAUUACAGAGAAAA UCGGCAGGGUUGGCAAA AUAGUAUUCGACAUAAU CUCAGUUUGAACGAUUG CUUCGUCAAGAUACCAC GCGACAAAAGUAAUCCU GACGAAGAGGAAGAACA CGGAGGGGCCGCAGGUA AAGGCAGUUAUUGGUGU CUCGAUCCAUCCGCCAA UGAAAUGUUUGAGCAUG GUAAUUACAGGCGUAGA	PD-FKH-F	TCGGGGAACAACC ATTTGGC	PD-FKH-R	CGGGACTCCGGGAAGATT G	96		This study

AGAACUCGCAAACAACG AGCCUUUUUACAGAGUA AAGAGGAAUCCCGAAGA UCUUUCUUACAACUUUU AGACGAUGAUCGAGGAA AUAUCGAGAGAUGGUGU GAUAGCGUUGAAAUUCG GAACACAGAGAACAAGA UAGAAAAGGAGAAGGAA GUGGAUUACGCGAGUGG UUCGGA							
**CaM** AUGGUACCAUAACUACC AAAGAAUUGGGCACCGU UAUGAGAUCUCUUGGUC AAAAUCCCACGGAAGCU GAGCUUCAAGAUAUGAU AAACGAAGUCGAUGCAG ACGGAAACGGUACCAUC GAUUUCCCGGAAUUUUU GACCAUGAUGGCACGUA AAAUGAAGGAUACUGAC AGCGAAGAGGAGAUAAG AGAGGCGUUCAGAGUGU UUGAUAAAGAUGGUAAU GGUUUCAUAUCUGCGGC AGAACUUAGACACGUCA UGACAAAUCUUGGAGAA AAGCUUACCGACGAAGA GGUAGACGAAAUGAU	PD-CaM-F	ACCAAAGAATTGG GCACCGT	PD-CaM-R	GGTACCGTTTCCGTCTGCA T	102	Primers necessarily overlap the dsRNA region	This study

Long A dsRNA - targeting genes VATPase and RPC2 (1306 bp)

AGGCUCUCAAGCUCGGUUUCGUUGCUGGAGUUAUACUACGUGAACGCAUUCCUGCAUUUGAACGCAUGCUAUGGCGUGCAUGUCGUGGAAAUGUAUU
UUUACGUCAAGCUGAAAUUGAAACACCCUUAGAGGAUCCUUCAACGGGUGACCAAGUCUUUAAAUCAGUAUUUAUUAUCUUCUUCCAAGGUGAUCAA
UUAAAGACGCGAGUUAAAAAAAUAUGCGAAGGUUUCAGGGCAACUUUAUAUCCCUGUCCUGAAGCACCAGCUGAUCGAAGGGAAAUGGCUAUGGGAG
UGAUGACACGUAUUGAAGAUUUAAACACGGUUCUUGGUCAAACUCAAGAUCAUCGUCACCGUGUUUUAGUAGCUGCAGCAAAAAAUAUCAAAAAUUG
GUUUGUUAAAGUACGAAAAAUAAAGGCAAUUUAAACCAGCGUAAAACUUGCUCUGGAACUCCACCCAAUGAAGACGAAGAGUAUGUAAGAAAGCUGA
AAGUCCUUCCAUUAGGACUAAAAUACCAACAGUUAAAACAGCCCAAAAUGCGAAUACUAUGCAAAGGAAAAUACCACCAGUCCAACCUUCACGAGUC
AAUCCAUUUCUCAUUACCAUAGCCCAUAAUACUUCUGAGAGUUGAGCAUGAGCAAGAGAUAAAGCCCACAAACGUAAAUAUGAUGCAGUAUGUGAAA
CACUACCAAGUACAUAUUCGAUAGUAUGAAUGCCUUGAUGUAUGAAAACUUCAGCCAUAUCUUCUUGUUCUUCUUUAUGACCACCUUGGACAACACC
UCCUGUUGGUUGCAUUGCACCAACAUUACCAUCUACAUCACCAUUUUCAGCACCAACGUUUGAUAGAAUAAUGGGUCCAUUAGUCGAUUCAAAUACA
AAAAAACCAGUAUGGAAGCACGAGAUCAUUGACAGCGAUGGGAUUGCUGCUCCGGGUGAAAUGGUAGAAAAUAGAAAAGUAAUGGUAAAUAAAUCU
ACGCCAUCCACCGCCGAUGUAGUUAAUCCUGCUAAUGCCCAAAAUGAAACAGAAUAUCGUGAUACUCCUCUUCUAUUCAAAGGUCCAGUUCCUGCUU
AUGUAGAAAAAGUUAUGAUUACUAGUAAUGCAGAAGAAGUAUUUUUAAUAAAGCUAUUGUUAAGACAAACUCGGAGACCUGAAGUAGGCGAUAAAU
UUAGUAGUCGUCACGGACAAAAAGGAGUAACGGGUUUAAUAGUCGAACAAGAAGAUAUGCCAUUUAACGAUUGUGGUAUAGUUCCUGAUAUGAUAA
UGAAUCCUCAUGGUUUUCCAUCCCGUAUGACUGUUGGAAAAUUACUAG

Long B dsRNA – targeting genes α-TUBULIN, APOPIN, RPA1 (1472 bp)

ACUGGAACAUACCGUCAGUUAUUCCAUCCAGAACAAUUGAUCACGGGCAAGGAAGAUGCUGCAAAUAAUUAUGCUCGUGGUCAUUACACUAUUGGUA
AAGAAAUCGUUGAUCUCGUUCUGGACCGUAUCCGAAAAUUGGCAGAUCAAUGUACUGGACUCCAAGGUUUUCUCAUCUUCCAUUCGUUCGGUGGUGG
AACCGGCUCUGGUUUCACUUCUCUCUUGAUGGAACGGCUUUCAGUAGACUACGGAAAGAAAUCGAAAUUAGAAUUUGCCAUCUAUCCAGCUCCACAA
GUCUCUACUGCUGUGGUUGAACCAUACAAUUCUAUCUUAACUACGCAUACCACGCUUGAACAUUCCGACUGCGCAUUUAUGGUUGACAACGAAGCUA
UUUAUGAUAUUUGUCGUCGUAAUUUGGACAUCGAAAGACCAACAUACACCAAUUUGAAUCGUUUGAUCGGUCAAAUCGUUUCUUCAAUCACUGCAUC
UCUACGAUUCGAUGUAACAGCUGAUGAAUUUCAUAGUAUCAUGGAAGUUCUUAGCUGGACUAGAUUAGGUUCUACUAUUUCUGGUCAACAAGAAUU
AAUUGAUAUUACGGUAGAACAAGCUGAACUUUCUGUGCCAUUUAAACACACAAAUGUUGAGCAAUGGAGCCGACUGAUACAAUGUAUCAAACAUGCU
CUUCCAUUCUUUAGUUCUCAAAUUGAUUCAUCAAGAUUUGUUUCAUAUAUCUGUAUUCAAGUUUUACCCCAUUUAUCAUUAAUUAAUACACCCGAUG
GAAGGGAUAGUCAGUUGGAAUUGUUAAAACUUCUUGCAGAAUUAACUGUAUUUUGCGGAACAAUCGAAAAUCCAGAAGAAAAAGUACAACAAUUAU
AUAACACACUAAUUACAUACAUGCCUCUUCCACCAGCCACAGAGAUAACGGAUGUCCCAAAGUUACAAUUCAGUCAUGUGGAAUGUUUAAUGUAUGC
UUUUCACAAAUUAUGUAAACAAACACCUGAUAGUGGACUUACAAUAGUUGCAGAAAAUAUUAACAGAAUGCGUUACAUGAGUCAUUUUCGUGCAAUC
CACAGAGGUUCAUUUUUCCAAGAAAUGAGAACUACAGAAGCAAGACAACUUUUACCAGAUGCUUGGGGAUUUAUUUGUCCUGUGCAUACACCAGACG
GUGCACCAUGUGGUUUACUUAAUCAUUUAACUAUGAAUUGCAUUGUUACAAAACAUCCAAAUGCCAAGUUAAAAGCUGCUAUACCAACGGUAUUAUU
AGAUCUUGGAAUGAUACCUAUUAAUUUUGUUGAUAAUUGGAAAGAUUCAUAUACUGUUUUAUUGGAUGGGAAAGUAAUUGGAGUUAUCGAUGAUAA
UACAAUAAAUAGAACCGUGGAUAAGUUAAGAGUACUCAAGAUAAAAGGUGAAGAGGUACCUCCUACAUUGGAAAUAGUAUUAAUUCCAAAGAGAAA
AGUACCAGCUCAAUAUCCAGG

Long C dsRNA – targeting genes INAPOP and NaKATPase (993 bp)

AACAUUUUUGAGCUAUCUUCACUGGAACUUGCACUAGAAUUUAAUUUAGCUGAAUCUUUAAUCUCUAAAUUGUUAUUUGAGUUGUCAUAUUUUGUG
CCAUUCGAAAUCUGUCCGUUUGAAGAUGAUUGAAUUUUGUCAACAAACAUUGGUAAAUUCAUCUGCAUUGUUUCCUCUCGUUCCUUUAUCAUAUUCA
CAAAUUCUUGUCCUUUCACCAUUAACAAAUAAUAACAUUUGGAGAACCAUUUGGCAUGUUGUUCCCAUGGAUCAUCCUGAGGUUCCCAAUCUUCCAA
UCCUUCACCACAAUGAUAACACAAUGUUUGAUCACCUUUUCCUAUAUAGAAAAAUCCAGCAUUCGCUAGUUGAUCUUUCUUCUGUGGCAUUGAUUUA
GGCCAGGUAUCAAAAGUACGUAGUCUAGCAUCAUAACUUAUAUAUUCUGGAUGUACCGGUGGCUUUGGUUUUUCAAGUCCUAACGAUGCUAACUUGG
UAGUUGUUGGAUAGAUAACCCGGUGUAUUUGUAAAUGGGAAAUAAUAUCCAGGAAAUCCACGACGUGGUAUAUAUUGAAUAGCACCCAUAUUUUCA
AUAUCAGCUGGAUUUUCUCCGGCACAGGAUACCCAAACAGUGUCUAGUCUUUGACUUUGAGCACUUUCUUCCUUCUUGAUAUGAUCUCGAAGAUCAG
UUGGCAUACCAGGUGGGAGGUUUUUAGUGUCAUUAUAGAAAUUUGGCAUCCAUCCAAAGAUCUUAUUCAACUUGAGAAAAAUGCAAGGAGCCGAUU
UGUUGAACCCAUAUUUCCUCUCUUUCGUACAUUGUCCCCAGUGGGUCAUAUCAACGUCACAAACUUUACCAGGUGGCACGGUUUACCAUAAUCGCA
AUGAAUUCUUUGUUCAGCGUCAGUUCCAGACGCUCCAUCUUUUUGGUAUUCUUGCAAAAAUGCAUCCAAUUCUUUGGUCCAAUGAAGAAAAUUACCU
UCGUCACUAGCUUUGUACCAGAUUAG

**RPC2**	PD-RNAPOL3-F	TGGTGAACCACTTGAAGCGT	PD-RNAPOL3-R	CGTTAGGACTGCTCTGGGTC	115		This study
**RPA1**	PD-RNAPOL1-F	GGCCAGCCGAAGATTTACCA	PD-RNAPOL1-R	GCTGCAGACTTTCCAGCCAT	127		This study
**VATPase**	PD-VATPase-F	ATTGGGTGGAGTTCCAGAGC	PD-VATPase-R	TCGGTAGTCGATTGAGCAGC	100		This study
**NaKATPase**	PD-NaKATPase-F	AGCCCTCTCGTTGCTGTTTT	PD-NaKATPase-R	ATGTACGGAACCACGCCTTT	120		This study
**APOPIN**	PD-APOPIN-F	GAAAAGCGCTTGGCATCTCA	PD-APOPIN-R	AGCTTGCTTCCTAATCGCCA	126		This study
**INAPOP**	PD-INAPOP-F	ACATGGTACTACACAAAGCCG	PD-INAPOP-R	GCAGGTGCACATTTAACGCA	132		This study

References

Cameron RC, Duncan EJ, Dearden PK. Stable reference genes for the measurement of transcript abundance during larval caste development in the honeybee. Apidologie. 2013;44(4):357-66. doi: 10.1007/s13592-012-0187-0.
