# Supplementary material for: Gene silencing for invasive paper wasp management: Synthesized dsRNA can modify gene expression but did not affect mortality
Source: PLoS One. 2023 Jan 3;18(1):e0279983. doi: 10.1371/journal.pone.0279983 (PMC9810182; doi:10.1371/journal.pone.0279983)

S1 Figure. Comparing the relative expression of calmodulin (CaM) gene present in the 'megacocktail' with the GFP dsRNA and the sugar water control. The qPCR primers unavoidably overlapped the dsRNA region for CaM. The RT-qPCR results clearly demonstrate CaM dsRNA consumption due to the apparent massive increase in CaM gene 'expression' due to the qPCR primers binding to the reverse-transcribed dsRNA rather than indicating a real increase in gene expression.

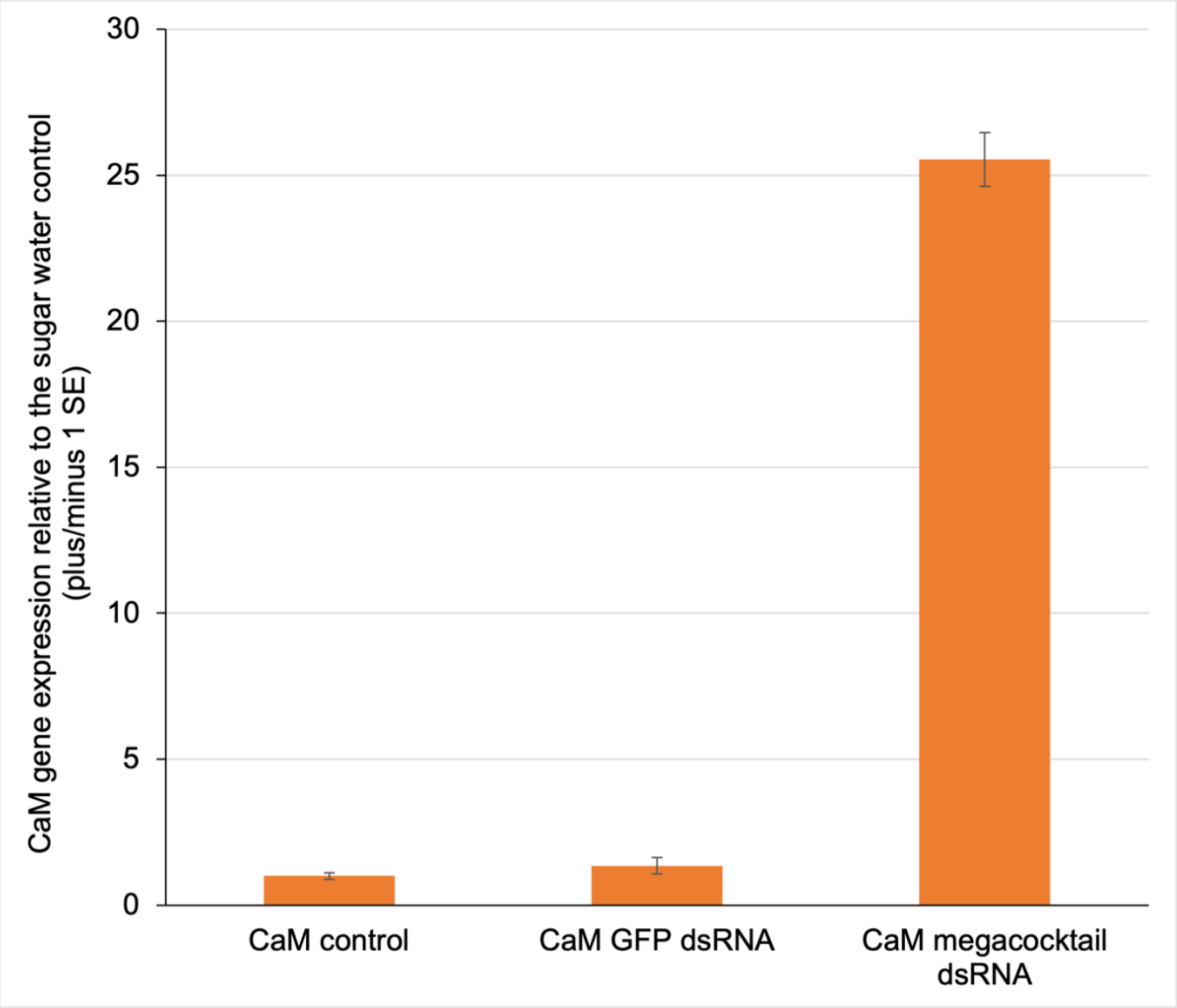

Supplement: S1 Fig — The qPCR primers unavoidably overlapped the dsRNA region for CaM. The RT-qPCR results clearly demonstrate CaM dsRNA consumption due to the apparent massive increase in CaM gene ‘expression’ due to the qPCR primers binding to the reverse-transcribed dsRNA rather than indicating a real increase in gene expression. (PDF) [file pone.0279983.s004.pdf]
