# Supplementary material for: Gene silencing for invasive paper wasp management: Synthesized dsRNA can modify gene expression but did not affect mortality
Source: PLoS One. 2023 Jan 3;18(1):e0279983. doi: 10.1371/journal.pone.0279983 (PMC9810182; doi:10.1371/journal.pone.0279983)

S2 Figure. Survival of *P. dominula* forager wasps fed four different dsRNA gene targets at three different concentrations and in two presentations, naked dsRNA on the left or CQD-protected dsRNA shown on the right, for 10 days in captivity in the laboratory.

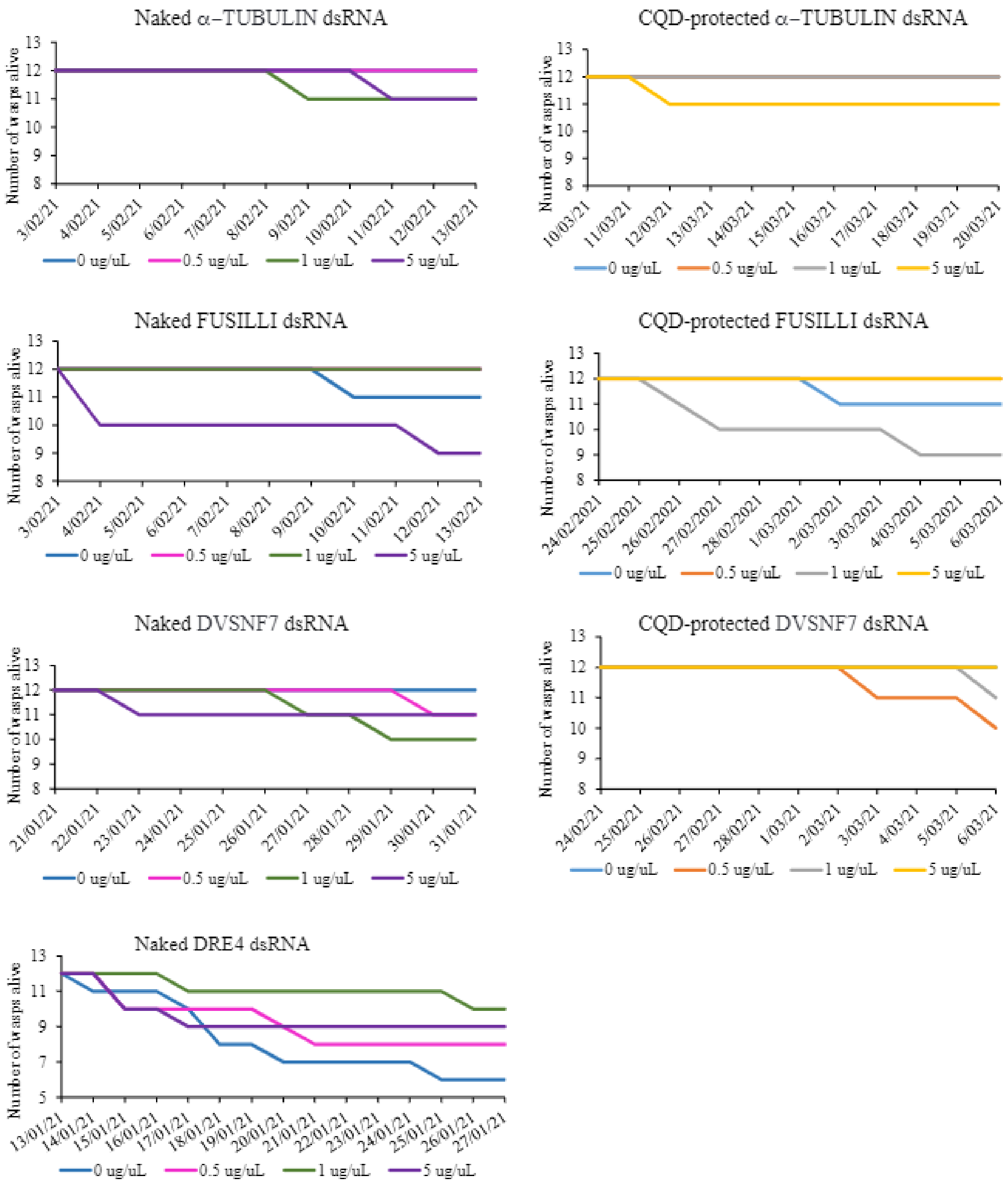

Supplement: S2 Fig — (PDF) [file pone.0279983.s005.pdf]
